# Bidirectional Expression of Metabolic, Structural, and Immune Pathways in Early Myopia and Hyperopia

**DOI:** 10.3389/fnins.2016.00390

**Published:** 2016-08-30

**Authors:** Nina Riddell, Loretta Giummarra, Nathan E. Hall, Sheila G. Crewther

**Affiliations:** ^1^Department of Psychology and Counselling, La Trobe UniversityMelbourne, VIC, Australia; ^2^Life Sciences Computation Centre, Victorian Life Sciences Computation InitiativeMelbourne, VIC, Australia; ^3^La Trobe UniversityMelbourne, VIC, Australia

**Keywords:** myopia, hyperopia, refractive error, transcriptome, RNA sequencing, gene expression, chick, retina

## Abstract

Myopia (short-sightedness) affects 1.45 billion people worldwide, many of whom will develop sight-threatening secondary disorders. Myopic eyes are characterized by excessive size while hyperopic (long-sighted) eyes are typically small. The biological and genetic mechanisms underpinning the retina's local control of these growth patterns remain unclear. In the present study, we used RNA sequencing to examine gene expression in the retina/RPE/choroid across 3 days of optically-induced myopia and hyperopia induction in chick. Data were analyzed for differential expression of single genes, and Gene Set Enrichment Analysis (GSEA) was used to identify gene sets correlated with ocular axial length and refraction across lens groups. Like previous studies, we found few single genes that were differentially-expressed in a sign-of-defocus dependent manner (only *BMP2* at 1 day). Using GSEA, however, we are the first to show that more subtle shifts in structural, metabolic, and immune pathway expression are correlated with the eye size and refractive changes induced by lens defocus. Our findings link gene expression with the morphological characteristics of refractive error, and suggest that physiological stress arising from metabolic and inflammatory pathway activation could increase the vulnerability of myopic eyes to secondary pathologies.

## Introduction

Myopia (short-sightedness) is the most common ocular disorder with rapidly increasing prevalence and severity worldwide (Seet et al., 2001; Pan et al., 2012). The increased axial length that characterizes myopic eyes results primarily from expansion of the fluid-filled vitreous chamber (Berman and Michaelson, 1964), and is accompanied by a decrease in the thickness of the vascular choroid (Hayes et al., 1986; Wallman et al., 1995; Westbrook et al., 1995) and thinning of fibrous scleral tissue that forms the eye's outer coating (Curtin and Teng, 1958). These structural changes greatly increase the risk of developing sight-threatening visual pathologies (Saw et al., 2005; Verhoeven et al., 2015), making the development of treatments to limit excessive ocular growth an important health and socioeconomic priority.

Ocular growth is controlled locally by the retina (Troilo et al., 1987; Wildsoet and Pettigrew, 1988) in a process that involves a complex interaction between an individual's genetic constitution and their environment (Wallman and Winawer, 2004). The role of the visual environment has been investigated using animal models in which rearing with negatively powered defocusing lenses or form deprivation occlusion increases the rate of growth (resulting in a larger myopic eye) while rearing with positively powered defocusing lenses slows growth (resulting in a smaller hyperopic eye; Wallman and Winawer, 2004). Microarray studies examining transcriptome changes in these models have implicated thousands of genes, however relatively few findings have been replicated across studies and it remains unclear which genes are important for controlling ocular growth (Ashby and Feldkaemper, 2010; Stone and Khurana, 2010; Stone et al., 2011).

Many of the transcriptome studies conducted to date have investigated a single ocular growth phenotype (e.g., myopia or hyperopia induction relative to controls; Tkatchenko et al., 2006; Brand et al., 2007; Mcglinn et al., 2007; Schippert et al., 2008). This experimental design makes it challenging to separate the genes involved in growth processes from those responding to the secondary effects of lens-wear (i.e., blur and physiological stress). Experimental designs that concurrently compare myopia and hyperopia induction enable identification of genes with expression profiles that are discriminatory for different ocular growth trajectories. Such genes are more likely to be directly involved in growth processes (thus providing attractive targets for therapy), and may also help to explain why myopic eyes are at a higher risk for secondary pathologies than their hyperopic counterparts. Although several studies have compared transcriptome-wide responses during ocular growth increases and decreases (Shelton et al., 2008; Ashby and Feldkaemper, 2010; Stone et al., 2011), few genes showing sign-of-defocus dependent expression have been identified and it has been suggested that distinctive (rather than bidirectional) genetic responses underlie myopia and hyperopia (Ashby and Feldkaemper, 2010; Stone et al., 2011).

Opportunities now exist to improve on the methods used by these past studies. Expression changes in animal models of refractive error have yet to be investigated using next generation RNA sequencing which is more quantitative, sensitive, and reproducible than the microarray technologies used previously (Wang et al., 2009). Additionally, most studies to date have analyzed data at the single gene level or used first generation pathway analyses to associate biological functions with lists of differentially-expressed genes (García-Campos et al., 2015). Second generation pathway analyses, such as gene set enrichment analysis (GSEA), are able to identify responses that are subtle at the single gene level because they do not require an arbitrary cut-off for differentially-expressed genes (Subramanian et al., 2005). Although the results of GSEA are more replicable and interpretable than single gene measures (Subramanian et al., 2005; Manoli et al., 2006), it has only been employed by one ocular growth study to date (Tkatchenko et al., 2006). Improvements can also be made at the experimental design level where very little is known about longitudinal changes because few studies have analyzed multiple treatment time-points (Brand et al., 2007; Mcglinn et al., 2007; Summers Rada and Wiechmann, 2009; Stone et al., 2011). Moreover, only one study has directly correlated expression shifts with the ocular growth phenotype of individual animals (Tkatchenko et al., 2006).

Thus, in the present study we used GSEA and a longitudinal design to identify gene expression patterns related to eye size and refraction across three conditions in chick: normal development, optically induced myopia, and optically induced hyperopia. As theories of visually regulated ocular growth hypothesize biological signals that propagate from the retina through to the choroid (e.g., Wallman et al., 1995; Rymer and Wildsoet, 2005; Crewther et al., 2006; Feldkaemper and Schaeffel, 2013), retina/RPE/choroid samples were profiled using RNA sequencing following 1, 2, and 3 days of lens-wear, or no lens rearing. We then used GSEA to identify gene sets correlated with ocular axial length and refraction across lens groups at each time-point.

## Materials and methods

### Animals and rearing

One hundred male chicks (Leghorn/New Hampshire), obtained from a commercial hatchery, were raised from post-hatch days 0–4 under a 12-h day/night light cycle (beginning at 8 a.m.) in groups of < 25. In the middle of the light cycle on day 5, chicks were randomly assigned to a lens condition (+10 or −10 diopters, or No Lens), and lenses (Polymethyl Methacrylate) attached to Velcro were fixed to the periocular feathers of the right eye. Following a further 1, 2, or 3 days with lenses attached, 10–12 chicks per lens group were anaesthetized (ketamine, 45 mg/kg; xylazine, 4.5 mg/kg i.m.) and right eye refraction and axial dimensions determined by retinoscopy (Keeler, Vista Diagnostic Instruments) and A-Scan ultrasonography (A-Scan III, TSL; Teknar, Inc. St Louis, USA; 7 MHz probe). Chicks were euthanized and their right eyes were enucleated. The retina/RPE/choroid was immediately collected from the posterior eyecup and frozen in liquid nitrogen before being transferred to −80°C. Note that tissue was collected from right eyes only to avoid the confounding influence of right/left eye developmental asymmetries in chick (Rogers and Bolden, 1991). Similarly, separate control animals were used because monocular treatments in chick can affect blood flow (Shih et al., 1993; Jin and Stjernschantz, 2000), refraction and axial length (Wildsoet and Wallman, 1995) in the contralateral eye. All procedures were conducted in accordance with the protocols approved by the La Trobe University Animal Ethics Committee and adhere to the ARVO Statement for the use of Animals in Ophthalmic and Vision Research.

### RNA isolation and library construction

Four chicks per lens*time condition were chosen for RNA extraction based on strong and cohesive biometric responses. The selected samples had been collected between 1 and 3 p.m. on days 1–3, and were counterbalanced for the order of collection across lens-groups. Total RNA was isolated from the retina/RPE/choroid using the miRNeasy Mini Kit (Qiagen, Germantown, MD, USA) including DNase digestion. RNA quality and quantity was assessed on the 2100 Bioanalyzer (RNA 6000 Nano Kit; Agilent Technologies, Santa Clara, CA, USA). All samples had an RNA integrity number (RIN) of >8.3. RNA quantity was also assessed on the Qubit 2.0 Fluorometer (RNA-HS assay; Life Technologies, Carlsbad, CA, USA).

Using an average of concentration measures obtained from Qubit and Bioanalyzer assays, 2.5 μg of RNA from each sample was used for library preparation and RNA sequencing. Libraries were prepared using the TruSeq Stranded mRNA LS kit (Illumina, San Diego, CA, USA) with dual indexing according to the manufacturer's instructions. The generated libraries were assessed on the 2100 Bioanalyzer (DNA 1000 kit; Agilent Technologies, Santa Clara, CA, USA) to ensure an average size distribution of approximately 280 bps, then quantified on the Qubit 2.0 Fluorometer (dsDNA HS assay; Life Technologies, Carlsbad, CA, USA) and by qPCR (GeneRead Library Quant Array; Qiagen, Germantown, MD, USA). Libraries were normalized to 10 nM in Tris-HCl (10 nM, pH8.5 with 0.1% Tween 20), pooled, and prepared for cluster generation on the Illumina cBot using the TruSeq SR Cluster Kit V3-cBot (Illumina, San Diego, CA, USA) with denatured template DNA diluted to 7 pM. The flow cell and sequencing reagents (TruSeq SBS Kit V3; Illumina, San Diego, CA, USA) were loaded on the Illumina HiSeq 1500 and a dual-index, single-end, 100 bp sequencing run performed. RNA-Seq data for each sample are available at the NCBI Gene Expression Omnibus under accession number GSE78042 (www.ncbi.nlm.nih.gov/geo/query/acc.cgi?acc=GSE78042).

### Biometric data analysis

Between group statistical comparisons of refraction and axial length were made using Analyses of Variance (ANOVA) with relevant *post-hoc* tests as required. Both dependent variables were normally distributed (Shapiro Wilks Test *p* > 0.05 and/or skewness and kurtosis within acceptable range), however refraction data violated the assumption of equal variances (Levene's Test *p* < 0.05). A conservative significance threshold (α = 0.001) was used for tests of group differences in refractive state to combat any resulting Type I error inflation (Harwell et al., 1992).

### Sequencing data analysis

#### Pre-processing

Three samples were sequenced in the 1 and 2 day No Lens groups, and 4 samples were sequenced in all other conditions. Read quality was assessed using FastQC (www.bioinformatics.bbsrc.ac.uk/projects/fastqc/), and adapter and low quality (Q-score < 10) sequences removed using CutAdapt (Martin, 2011) and Trimmomatic (Bolger et al., 2014). Reads were mapped to the chick genome (GalGal4) using Tophat2 (Kim et al., 2013) and Bowtie2 (Langmead and Salzberg, 2012). The number of reads uniquely mapping to each gene was counted using existing gene models with HTSeq (Anders et al., 2014). Supplementary Table S1 lists the total number of gene counts for each sample, Supplementary Figure S1 shows the variance for each sample group, and Supplementary Figure S2 shows PCA plots for samples at each time-point.

#### Differential gene expression

Our initial single gene analysis using EdgeR (Robinson et al., 2010) identified a very large number of differentially-expressed genes following 3 days of positive lens-wear (relative to other conditions). At the suggestion of a reviewer, we subsequently reanalysed our data using the more conservative DESeq2 approach (Love et al., 2014). We chose to incorporate the latter DESeq2 results in the manuscript as our own comparisons indicated that this method was better able to discriminate genes with previously demonstrated relevance for the treatment factors of interest (myopia and hyperopia induction). Moreover, previous research suggests that DESeq2 is better able to control the Type I error rate (Soneson and Delorenzi, 2013). The results of the EdgeR analysis are provided in Supplementary Tables S2, S3.

Using DESeq2, we first assessed differential gene expression for each lens condition (myopia or hyperopia induction) relative to age matched no lens controls. Gene counts for all lens conditions were loaded into R (R Core Team, 2013) separately for each time-point (1, 2, and 3 days). Differentially expressed genes (DEG) were identified using the SARTools (Varet et al., 2016) DESeq2 (Love et al., 2014) pipeline with default settings and a Benjamini-Hochberg adjusted *p*-value cut-off of 0.05. DEG in each lens condition were then tested for over-representation of Gene Ontology (GO) level 3–5 terms in ConsensusPathDB (Kamburov et al., 2009) using all genes measured as the background (FDR *q* < 0.05). To facilitate interpretation of GO results, the Cytoscape Enrichment Map app (Merico et al., 2010) was used to cluster over-represented ontologies containing similar genes. Ontologies that clustered together with an overlap co-efficient of >0.55 were collapsed into a single annotation and visualized as chord diagrams using the GOplot package in R (Walter et al., 2015). Results from the original ConsensusPathDB analysis (i.e., before redundant annotations were combined) are provided in Supplementary Tables S4, S5.

Differential gene expression was also assessed across time within each lens group. Gene counts for all time-points were loaded into R (R Core Team, 2013) separately for each lens condition. Genes differentially expressed in each lens group (negative, positive, and no lens) between 1–2 and 2–3 days were identified. All other processes were conducted as described above for cross-lens comparisons. Hypergeometric tests (*p* < 0.05) were used to determine whether the overlap in gene findings within and across groups was more than expected as a result of chance (using the total number of genes measured as the reference).

#### Validation of single-gene findings using previously published data

Rather than performing qPCR validation for a small number of DEG (Hughes, 2009), we chose to use GSEA to validate our single gene results against a previously published microarray dataset of similar design. This approach was based on the reasoning that the genes classified as significantly up- and down-regulated in our study should be enriched at the top and bottom, respectively, of a microarray dataset testing the same treatment effect (Suárez-Fariñas et al., 2010).

The most similar available microarray data series was obtained from the GEO Database (GSE24641). In this microarray study, Stone et al. (2011) assessed the effects of 6 h and 3 days of negative and positive lens-wear relative to contralateral control eyes in chick. A comparison of Stone's methods with those of the present study is provided in Supplementary Table S6. The raw microarray CEL files were pre-processed using robust multi-array analysis (RMA), probe sets were median summarized, and log intensity values were imported into the GSEA program. We tested Stone's 6 h and 3 day negative lens data for enrichment of the genes up-regulated and down-regulated in the present study following 1, 2, and 3 days of negative lens-wear using the Signal2Noise metric. We expected that this approach would validate genes that respond robustly to negative lens-wear under the varied conditions encompassed by the two datasets, and help to rule out the influence of small undesirable methodological differences (e.g., contralateral eye effects, exact light intensity, space in the rearing cage, goggle material etc.) likely to be reproduced by a within-lab qPCR validation. The genes differentially-expressed following positive lens-wear were not validated using this approach because GSEA cannot accurately adjust the enrichment statistic for very small gene set sizes (Subramanian et al., 2005).

#### Gene set enrichment analysis

GSEA (Subramanian et al., 2005) was used to analyse the expression of Kyoto Encyclopaedia of Genes and Genomes (KEGG) pathways from the molecular signatures database (mSigDB) (Kanehisa and Goto, 2000; Liberzon et al., 2011). The primary analyses were designed to identify KEGG gene sets correlated with ocular axial length and refraction across lens groups at each time-point. Expression values (counts per million) were imported into the GSEA program (Subramanian et al., 2007). GSEA was conducted with 1000 phenotype permutations using a continuous increasing phenotype label based on the axial length or refraction measure for each sample across lens groups at each time-point (1, 2, and 3 days). Pearson's metric, which uses Pearson's correlation to determine the degree of linear relationship between the gene set and expression profiles, was used for ranking genes. An FDR cut-off of 0.25 was used (as recommended by the Broad Institute GSEA User Guide; http://software.broadinstitute.org/gsea/doc/GSEAUserGuideFrame.html) and gene set sizes limited to 15–500. As with the single gene measures, further GSEA were conducted to assess KEGG pathway expression changes across time within each lens group. A continuous increasing phenotype label was used based on time in hours for each sample. All other processes were as conducted as described above for cross lens comparisons.

#### Leading edge subset analysis and enrichment map figure generation

Following GSEA, leading edge subset (LES) analysis was used to identify the most relevant genes within each enriched pathway (these LES genes are referred to as “core” pathway genes in the results section). This additional analysis allowed identification of enriched gene sets representing similar biological signals (i.e., gene sets with highly similar core genes; Subramanian et al., 2005). To cluster these redundant pathways we used the Enrichment Map App (Merico et al., 2010). For pathways implicated in both refraction and axial length analyses, the LES genes were combined into a single list. A gene set file was then constructed from the LES of each enriched pathway and imported into Cytoscape along with the GSEA results. For each time-point (1, 2, and 3 days), an enrichment map was built using an overlap coefficient cut-off of 0.3. In the resulting network diagrams, each node represents a pathway. Node size indicates the number of core genes in the LES for that pathway, and connections between nodes indicate common LES genes. To further interrogate the basis of pathway correlations with eye size, we also created line graphs showing the mean log2-fold change for each pathway's LES genes across lens groups.

## Results

### Ocular refraction and axial dimensions

**Figure 1:**
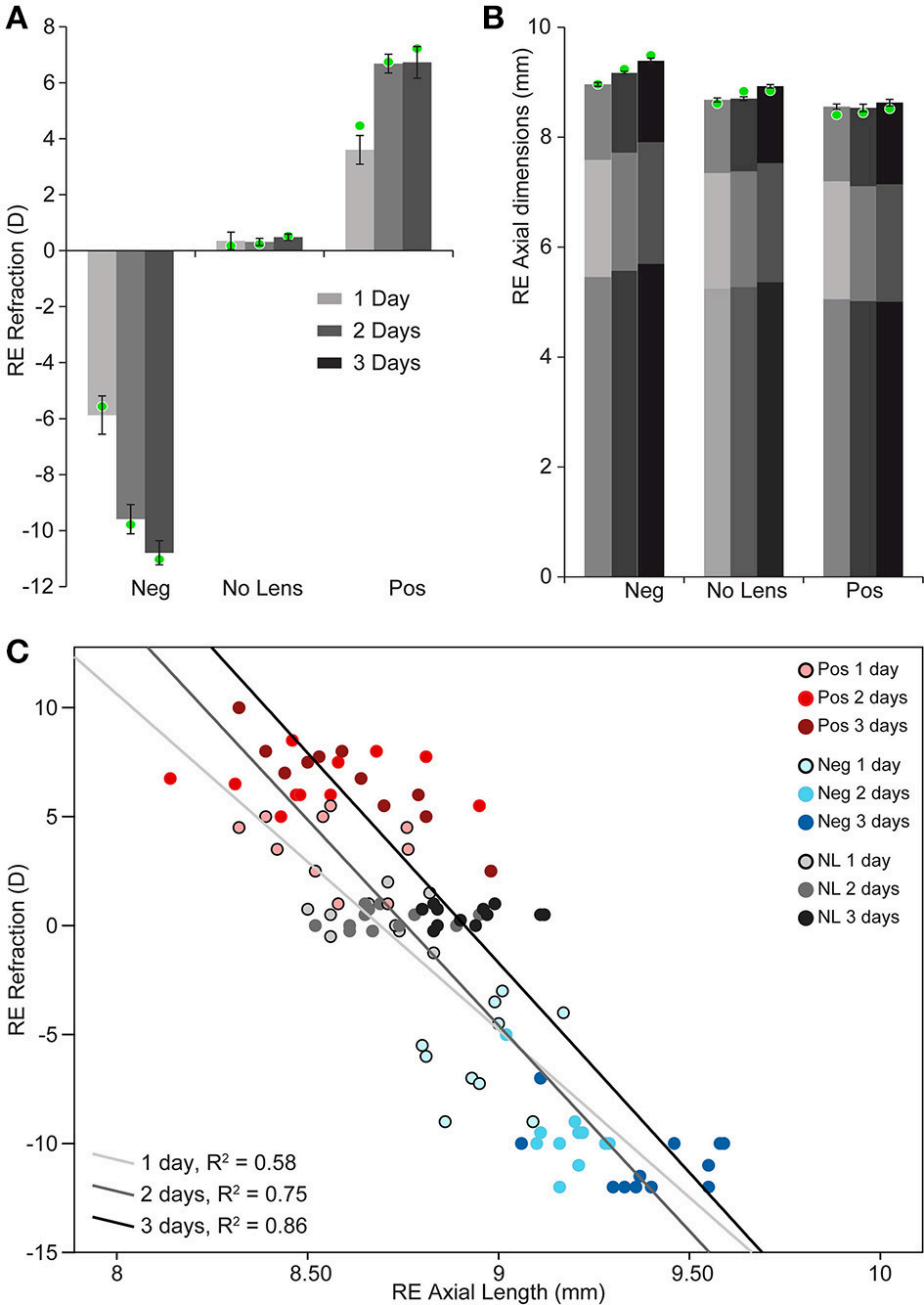
**Graphs showing refraction and axial dimensions during myopia and hyperopia induction, and normal development. (A)** Mean (±SE) right eye refraction. **(B)** Mean (±SE) right eye axial length (measured from the cornea to the outer limiting membrane of the retina). Mean anterior and vitreous chamber depths are shown as darker shaded regions at the top and bottom, respectively, of axial length measures. These biometric values are for *n* = 10–12 chicks per condition. Mean refraction and axial length for the subset of these chicks chosen for sequencing are shown as green circles superimposed on the graphs in “A” (*n* = 4 per condition selected for strong and cohesive phenotypic responses). **(C)** Scatter plot showing the relationship between refractive error and axial length (Pearson's correlation coefficient for all data points: *r* = −0.846, *p* < 0.001).

Chicks wearing −10D lenses became myopic and those wearing +10D lenses became hyperopic (Figure 1A). This refractive compensation was accompanied by an increased (−10D) or decreased (+10D) rate of axial growth relative to normally developing eyes (Figure 1B). As expected, there was a strong negative correlation between axial length and ocular refraction (Figure 1C).

Two-way ANOVAs were conducted to compare the effects of lens-wear (+10D, No Lens, −10D) and induction time (1, 2, 3 days) on refraction and axial length. There was a significant main effect of lens-wear [*F*_(2, 90)_ = 842.96, *p* < 0.001], but not time [*F*_(2, 90)_ = 1.24, *p* = 0.296], on ocular refraction. A significant interaction between lens-wear and time was also observed [*F*_(4, 90)_ = 24.42, *p* < 0.001]. *Post-hoc* tests revealed that the refractive state of all lens groups was significantly different by the earliest 1 day induction time-point (*p* < 0.001 for all comparisons). In contrast, the main effects of lens-wear [*F*_(2, 91)_ = 134.52, *p* < 0.001] and time [*F*_(2, 91)_ = 24.18, *p* < 0.001] on ocular axial length were both significant. As expected, an interaction effect was also observed [*F*_(4, 91)_ = 4.08, *p* = 0.004]. *Post-hoc* tests revealed that chicks wearing −10D lenses had longer axial lengths than No Lens controls by 1 day (Tukey HSD *p* < 0.001), however the axial length difference between +10D and No Lens chicks did not reach significance until 2 days (Tukey HSD *p* = 0.035). Because positive lens-wear slows axial elongation relative to normal development, the axial length difference between positive and No Lens groups (and thus the absolute effect size) is limited by the rate of growth in the No Lens group. This may explain why the axial length difference between No Lens and positive lens chicks was not significant on day 1.

### Genes differentially-expressed between lens groups at each time-point

**Figure 2:**
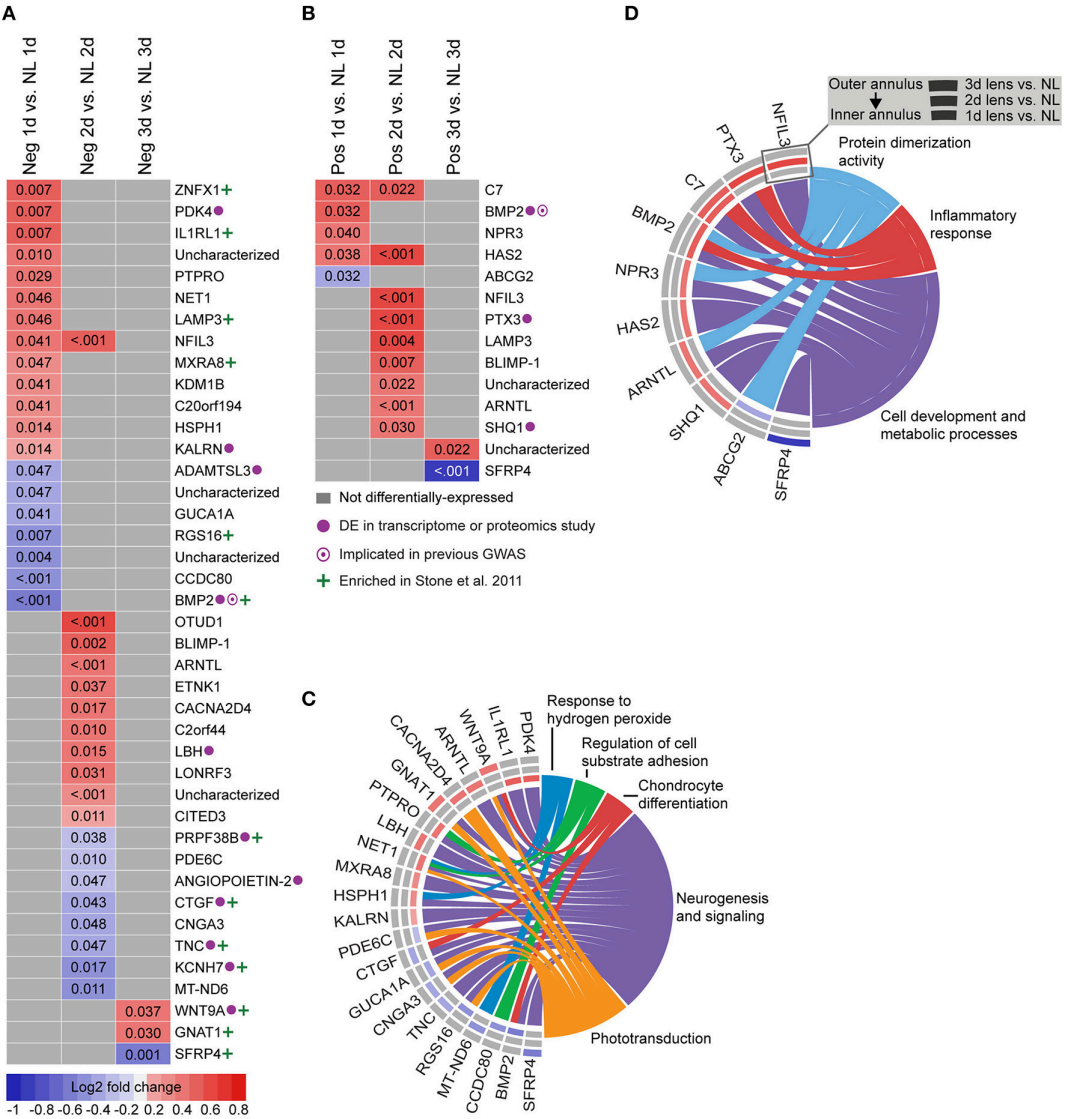
**Differential expression of single genes in lens relative to no lens groups. (A)** Heatmap showing genes differentially expressed following 1, 2, and 3 days of negative lens induced myopia induction. **(B)** Heatmap showing genes differentially expressed following 1, 2, and 3 days of positive lens induced hyperopia induction. Fold change is indicated by grid color (red = up-regulation, blue = down-regulation, gray = not differentially-expressed) and Benjamini–Hochberg adjusted *p*-values are super-imposed on the grid. Purple symbols indicate genes implicated in past exploratory animal or human studies of refractive error. Green symbols indicate genes enriched at the top or bottom of a comparable microarray dataset (see Supplementary Figure S3 for details) **(C)** Chord diagram showing over-represented GO terms for the genes differentially-expressed during myopia induction (i.e., all genes shown in “A”). **(D)** Chord diagram showing over-represented GO terms for the genes differentially-expressed during hyperopia induction (i.e., all genes shown in “B”). For chord diagrams, over-represented GO terms are shown on the right and genes contributing to this over-representation are shown on the left. Squares following gene symbols indicate whether a gene was differentially-expressed following 1, 2, or 3 days of lens-wear. For details of differential gene expression see Supplementary Table S7. For details of commonalities with past studies see Supplementary Table S8.

To provide a basis for comparison with past studies, we first assessed differential gene expression in lens groups relative to no lens controls. In the negative lens-group, 20, 19, and 3 genes were differentially-expressed at 1, 2, and 3 days, respectively (Figures 2A,C). In the positive lens condition, 5, 9, and 2 genes were differentially-expressed at 1, 2, and 3 days, respectively (Figures 2B,D). These single gene results demonstrated good concordance with past microarray studies; many of the DEG have been implicated previously (see Supplementary Table S8 for details), and the DEG following negative lens-wear were enriched at the top and bottom of the most similar available microarray dataset as assessed using GSEA (see Supplementary Figure S3). Notably only one gene, *BMP2*, was differentially expressed during both myopia and hyperopia induction in a sign-of-defocus dependent manner.

### Genes differentially-expressed within lens groups over time

**Figure 3:**
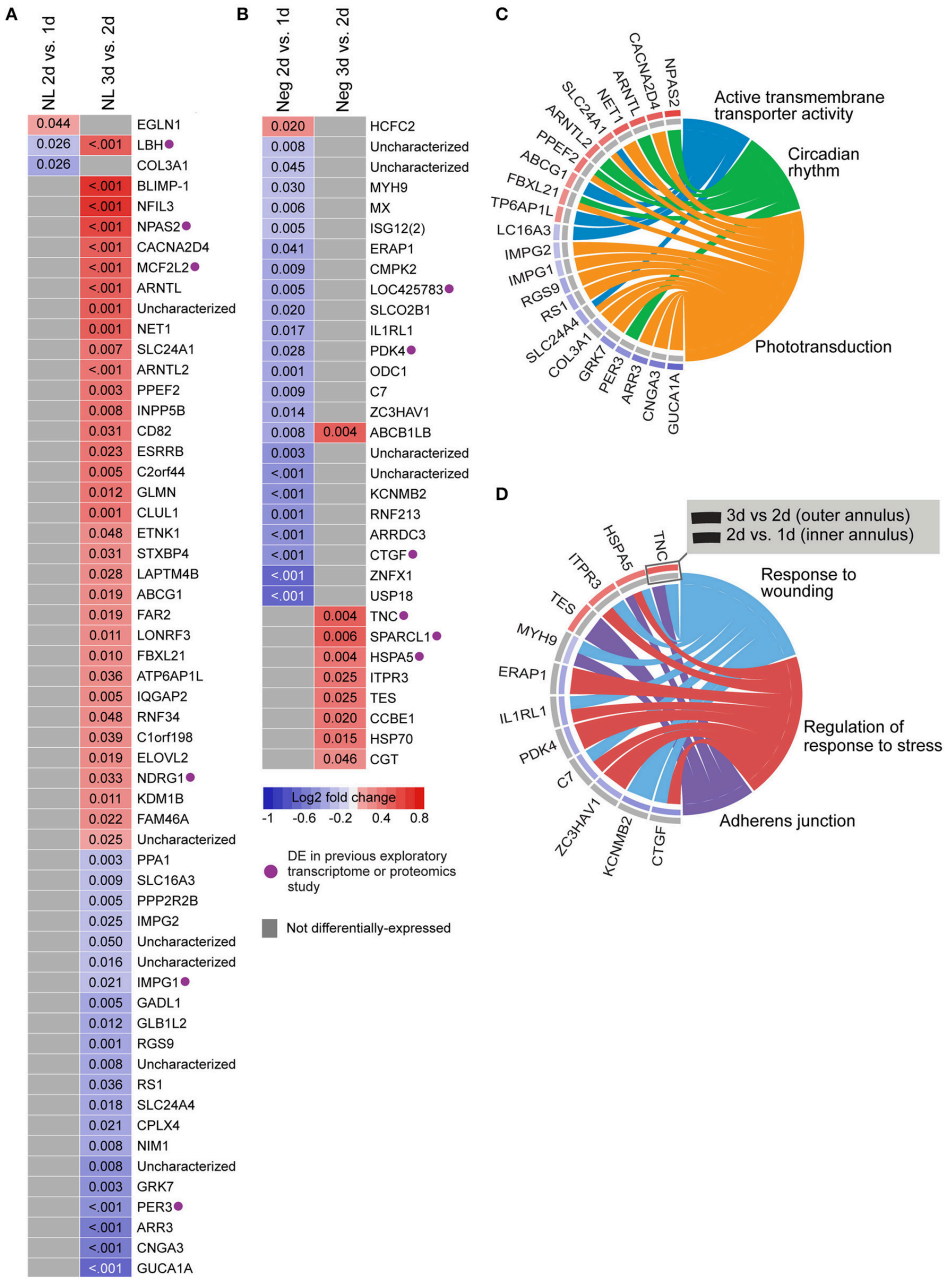
**Differential expression of single genes over time within No Lens and negative lens groups. (A)** Heatmap showing genes differentially expressed between 1–2 and 2–3 days of normal No Lens development. **(B)** Heatmap showing genes differentially expressed between 1–2 and 2–3 days of negative lens induced myopia induction. Note that no genes were differentially-expressed over time during hyperopia induction. Fold change is indicated by grid color (red = up-regulation, blue = down-regulation, gray = not differentially-expressed), and Benjamini–Hochberg adjusted *p*-values are super-imposed on the grid. Purple symbols indicate genes implicated in past exploratory animal studies of refractive error. **(C)** Chord diagram showing over-represented GO terms for the genes differentially-expressed over time during normal development (i.e., all genes shown in “A”). **(D)** Chord diagram showing over-represented GO terms for the genes differentially-expressed over time during myopia induction (i.e., all genes shown in “**B**”). For chord diagrams, over-represented GO terms are shown on the right and genes contributing to this over-representation are shown on the left. Squares following gene symbols indicate whether a gene was differentially-expressed between 1–2 and 2–3 days. For details of differential gene expression see Supplementary Table S9. For details of commonalities with past studies see Supplementary Table S8.

We also analyzed gene expression over time within each lens group (i.e., between days 1–2 and 2–3; see Supplementary Table S9 for detailed results). No genes were differentially-expressed over time during hyperopia induction. This may be because expression measures began at 1 day when substantial refractive compensation had already occurred. By comparison, 32 genes were differentially-expressed during myopia induction (Figures 3B,D), and 57 genes were differentially-expressed during normal development (Figures 3A,C).

**Figure 4:**
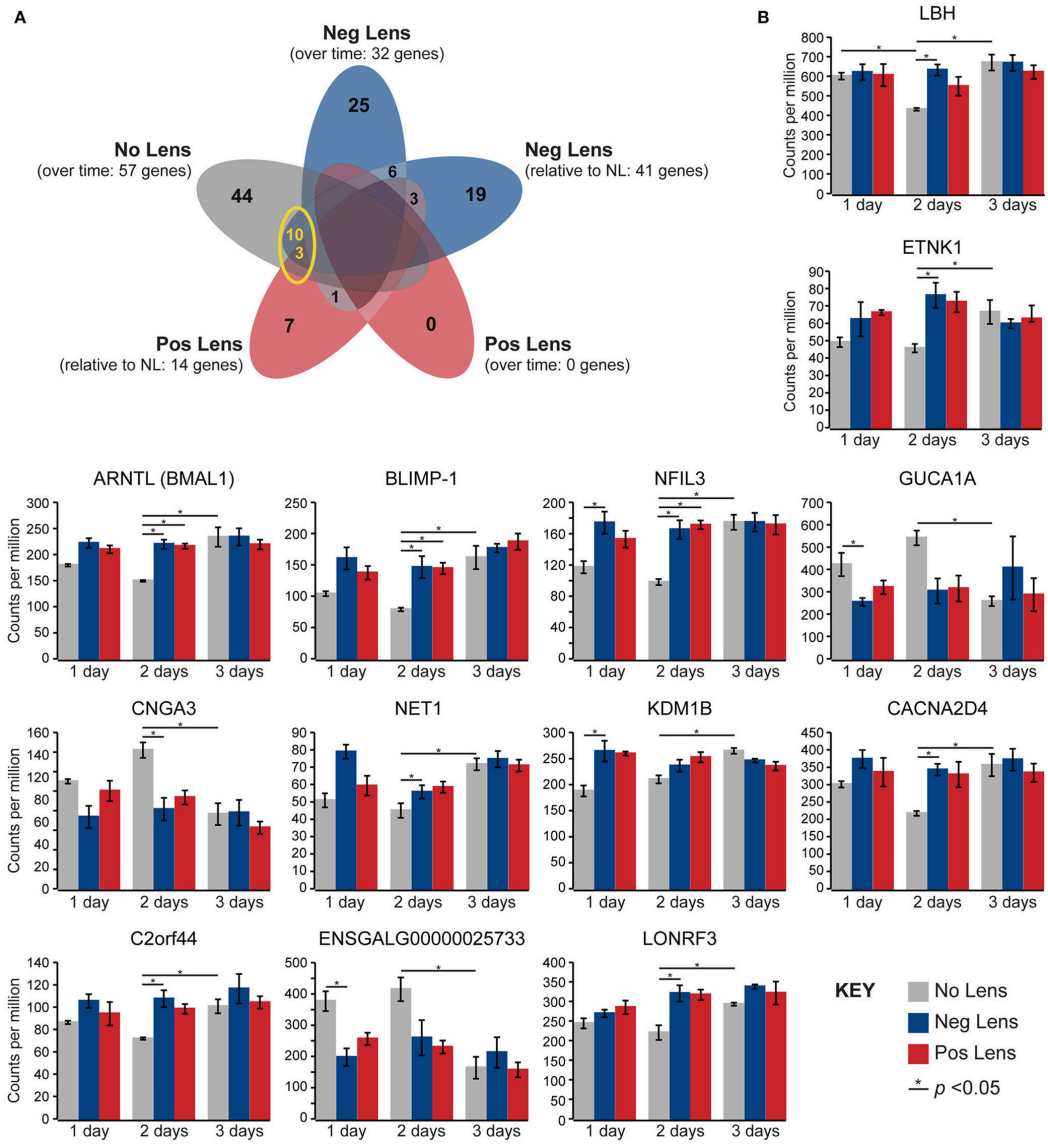
**Lens wear accelerates the time-course of developmental shifts in circadian and phototransduction gene expression. (A)** Venn diagram highlighting the number of genes that were differentially expressed across multiple conditions (i.e. overlap in gene findings between the within and across-group conditions shown in Figures 2A,B, 3A,B). The yellow circle highlights overlap in the genes differentially expressed over time in the no lens group, and the genes differentially expressed in lens vs. No Lens comparisons at 1, 2, or 3 days. This overlap suggests that perturbation of developmental expression changes could drive some of the differences seen when comparing normal development with refractive error phenotypes. **(B)** Column charts showing expression (counts per million, ±SE) of each gene highlighted in **(A)**. Note that, for most genes, lens wear appears to accelerate the time-course of developmental expression changes.

Normal developmental expression shifts were not seen in either lens group during the experimental period. There were, however, 13 commonalities between the genes differentially expressed across normal development and the genes differentially expressed when comparing lens and no lens groups at each time-point (Figure 4A). This degree of overlap in differentially-expressed gene findings with normal development was more than expected as a result of chance for both negative (*p* < 0.001) and positive (*p* < 0.001) lens groups. For most of the commonalities, lens wear appeared to accelerate the time-course of developmental expression shifts (Figure 4B). Genes showing these accelerated expression patterns in both negative and positive lens-groups were involved in circadian (*NFIL3, ARNTL*) and phototransduction (*BLIMP-1*) processes. An additional four phototransduction-related genes displayed accelerated expression patterns in the negative lens-group only (*GUCA1A, CNGA3, NET1, CACNA2D4*; see also Figure 3C). These findings suggest that lens wear (of both signs) perturbs the timing of developmental changes in circadian and phototransduction gene expression.

### Pathways correlated with eye size and refraction

In addition to assessing single gene changes, we used GSEA to identify pathway expression patterns related to eye size and refraction at each time-point across the three growth conditions (normal development, myopia induction, and hyperopia induction). Fourteen pathways were implicated in both refraction and axial length analyses. An additional 12 pathways were significantly associated with either axial length or refraction (Supplementary Table S10). For comparison, we also analyzed pathway expression changes over time within each lens group (Supplementary Table S11; note that because expression data were collected across post-hatch days 6–8 only, interpretation of within group results is limited by the lack of a common “0 h” starting measure on post-hatch day 5).

**Figure 5:**
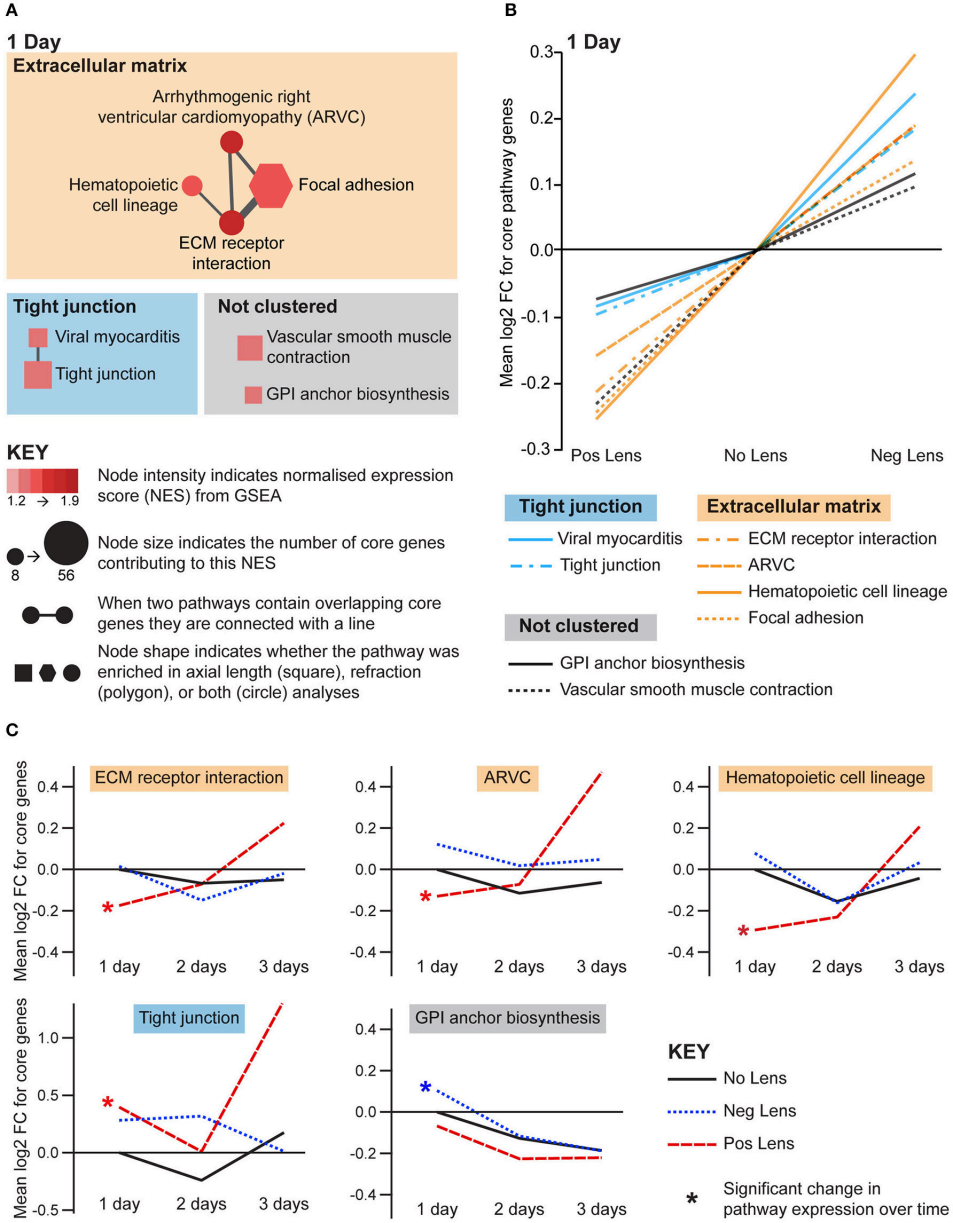
**Structural pathway expression changes during refractive compensation, and normal development. (A)** Network diagrams showing structural pathways positively correlated with eye size on day 1. Each node represents a pathway, and nodes are connected with a line when two pathways contain common core genes (representing related biological processes; thicker lines = more overlap). Node color indicates the direction of expression change (red = positive correlation with axial length and/or negative correlation with refraction). Node intensity indicates the normalized expression score (NES) from the axial length analysis (circle and square nodes) or the refraction analysis (polygon nodes). **(B)** Line graph showing mean log2-fold change for the core genes responsible for each pathways' enrichment. Note that this graph indicates that enrichment results were driven by roughly proportional expression shifts in both lens groups. **(C)** Structural pathways from “A” also showing expression changes across time within lens groups. Line graphs show the mean log2-fold change for core genes relative to the 1 day No Lens group. Note that because the core genes responsible for pathway enrichment vary for within and across group analyses, these values differ from those shown in “B”. For full details of GSEA results see Supplementary Tables S10, S11.

Structural pathways were positively correlated with eye size and negatively correlated with refraction on day 1 (Figures 5A,B), but not at later time-points. This agrees with biometric measures (Figure 1A) where much of the growth and refractive compensation occurred within the 1st day of lens wear. Several of the identified pathways could be clustered based on common core genes and biological functions (Figure 5A). A cluster of pathways related to extracellular matrix structure was implicated in both axial length and refraction analyses. In addition, tight junction, GPI (glycosylphosphatidylinositol) anchor biosynthesis, and smooth muscle contraction pathways were positively correlated with axial length only. Many of these structural pathways were also up-regulated over time in the positive lens group (following their initial down-regulation; Figure 5C), further emphasizing that expression levels depend on the stage of refractive compensation (i.e., early time-points when refractive compensation is rapid vs. later time-points when refractive compensation is almost complete).

**Figure 6:**
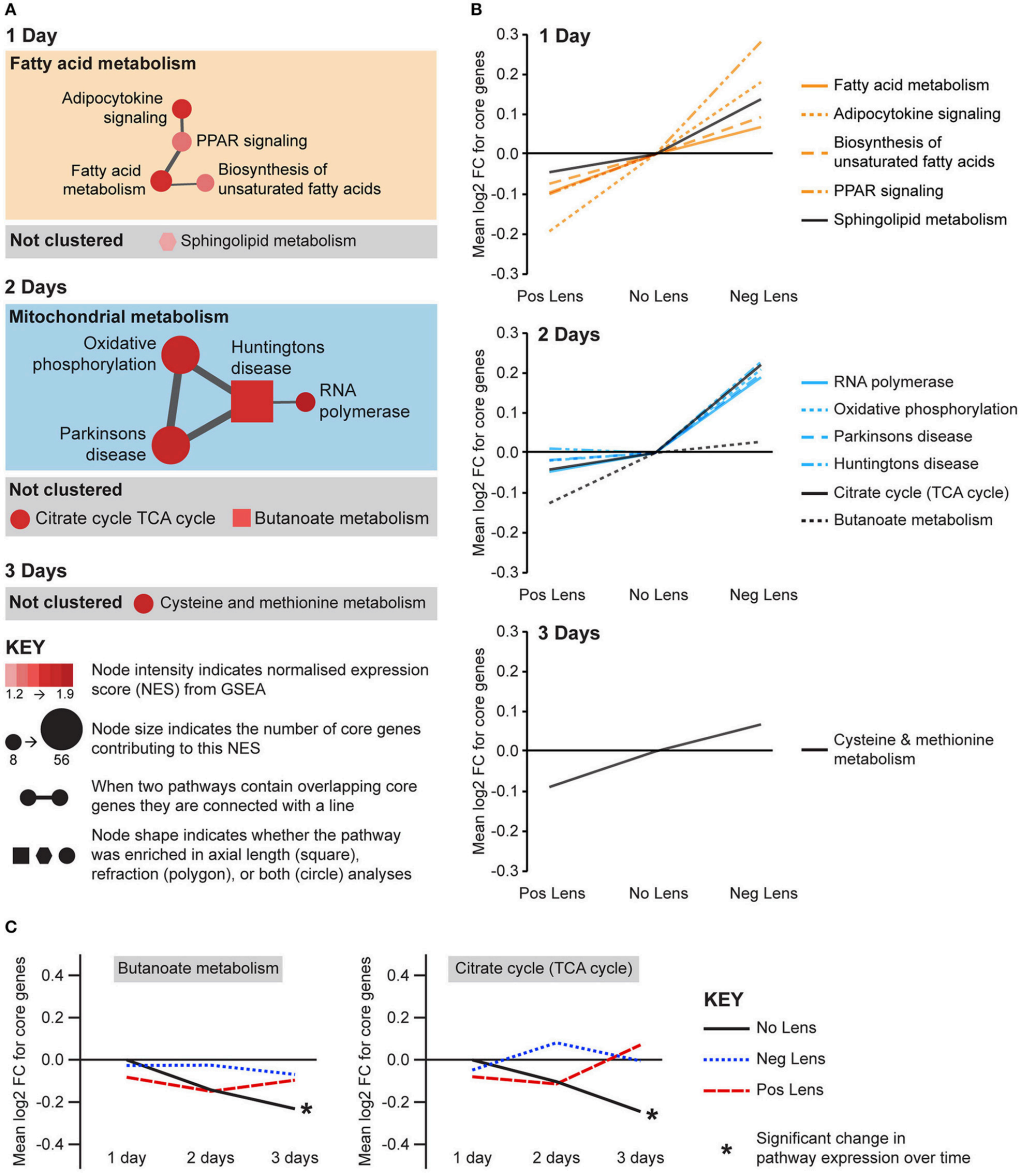
**Metabolic pathway expression changes during refractive compensation, and normal development. (A)** Network diagrams showing metabolic pathways positively correlated with eye size on days 1, 2, and 3. Each node represents a pathway, and nodes are connected with a line when two pathways contain common core genes (representing related biological processes; thicker lines = more similarity). Node color indicates the direction of expression change (red = positive correlation with axial length and/or negative correlation with refraction). Node intensity indicates the normalized expression score (NES) from the axial length analysis (circle and square nodes) or the refraction analysis (polygon nodes). **(B)** Line graph showing mean log2-fold change for the core genes responsible for each pathways' enrichment. Note that this graph indicates that enrichment results on day 2 were primarily driven by changes in one of the two lens groups. **(C)** Metabolic pathways from “A” also showing expression changes across time within lens groups. Line graphs show the mean log2-fold change for core genes relative to the 1 day no lens group. Note that because the core genes responsible for pathway enrichment vary for within and across group analyses, these values differ from those shown in “B.” For full details of GSEA results see Supplementary Tables S10, S11.

Eight metabolic pathways were positively correlated with eye size and negatively correlated with refraction, and an additional 3 pathways were uniquely associated with either axial length or refraction (Figure 6A). The earliest metabolic processes implicated were fatty acid and sphingolipid metabolism on day 1. These pathways all showed bidirectional expression responses (core genes demonstrated roughly proportional up-regulation in the negative lens group and down-regulation in the positive lens group; Figure 6B). By 2 days expression shifts were evident in pathways downstream of fatty acid metabolism (Figure 6A), however responses diverged across the two lens groups. The citrate (TCA) cycle pathway and a cluster of pathways related to mitochondrial metabolism were strongly up-regulated in the negative lens group, while the butanoate pathway (with core genes primarily related to ketogenesis) was strongly down-regulated in the positive lens group (Figure 6B). The citrate cycle pathway was also down-regulated over time within the No Lens group (Figure 6C). The higher expression of genes from this pathway in the negative lens group at 2 days appeared to result from a failure to follow this normal trajectory of downregulation.

**Figure 7:**
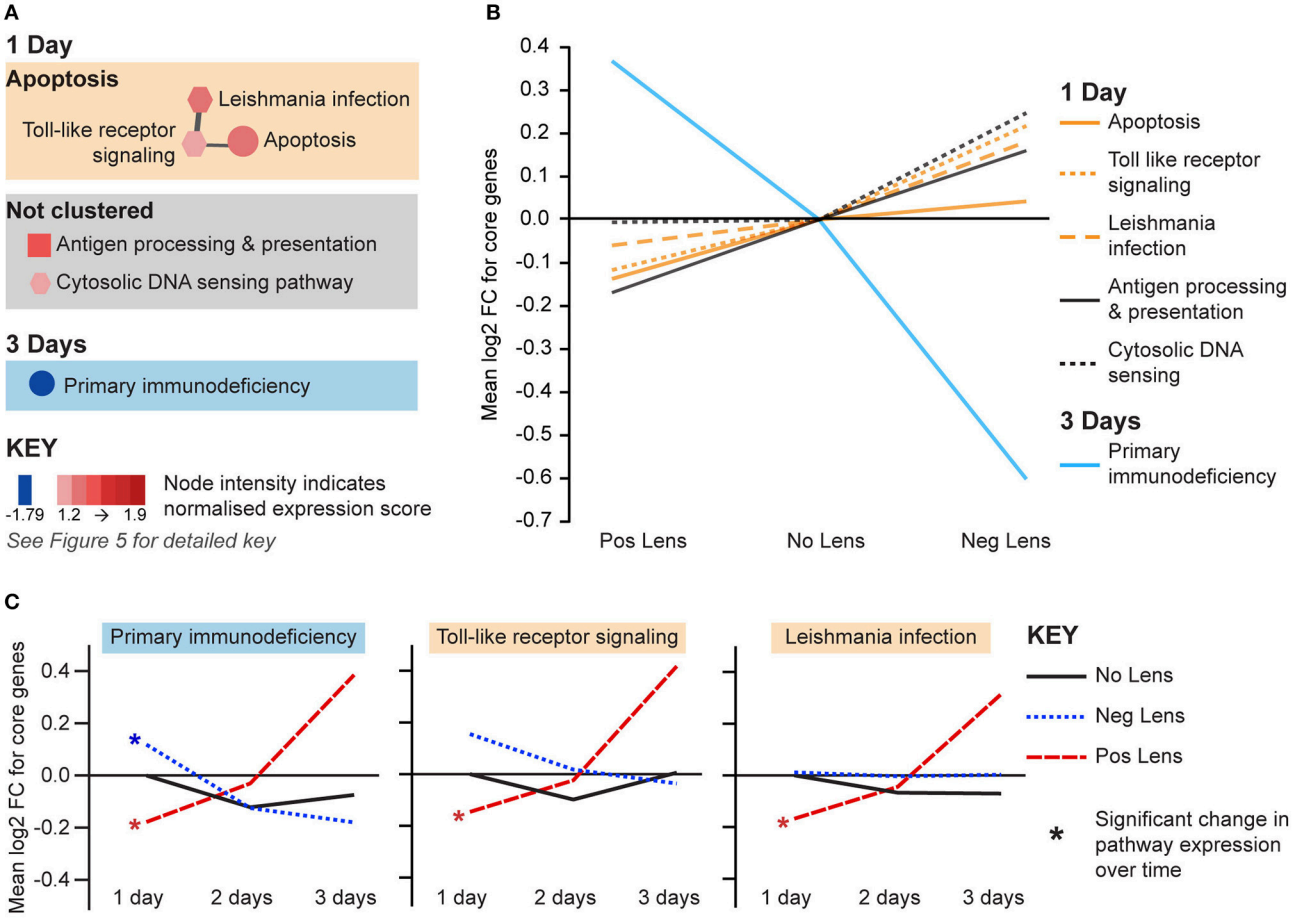
**Immune and apoptosis pathway expression changes during refractive compensation. (A)** Immune and apoptosis pathways correlated with eye size on days 1 and 3. These pathways are not clustered as the core gene sets responsible for enrichment were unique at each time-point. Node color indicates pathway normalized expression score (NES). Node color indicates the direction of expression change (red = positive correlation with axial length and/or negative correlation with refraction, blue = negative correlation with axial length and/or positive correlation with refraction). Node intensity indicates the normalized expression score (NES) from the axial length analysis (circle and square nodes) or the refraction analysis (polygon nodes). **(B)** Line graph showing mean log2-fold change for the core genes responsible for each pathways' enrichment. **(C)** The primary immunodeficiency pathway from “A” which also showed expression changes across time within positive and negative lens groups. This line graph shows the mean log2-fold change for core genes relative to the 1 day no lens group. Note that because the core genes responsible for pathway enrichment vary for within and across group analyses, these values differ from those shown in “B”. For full details of GSEA results see Supplementary Tables S10, S11.

The remaining pathways correlated with eye size and refraction were related to apoptosis and immune processes (Figure 7A). Apoptosis and immune-related pathways were positively correlated with axial length and negatively correlated with refraction on day 1 (Figure 7A). By day 3, a further immune pathway (primary immunodeficiency) was negatively correlated with axial length and positively correlated with refraction (Figures 7A,B). Additionally, the primary immunodeficiency pathway was up-regulated over time in the positive lens group and down-regulated over time in the negative lens group (the only pathway to show such an expression pattern in the present study; Figure 7C). Two pathways from the apoptosis cluster were also up-regulated over time within the positive lens group. These within and across group GSEA results suggest that the profile of immune and apoptosis pathway expression may reverse across the time-course of refractive compensation (particularly for chicks wearing positive lenses).

## Discussion

It has previously been suggested that distinct rather than bidirectional genetic mechanisms underlie eye growth increases during negative lens wear and growth slowing during positive lens wear (Ashby and Feldkaemper, 2010; Stone et al., 2011). However, we show here that gene expression across a range of structural, metabolic, and immune pathways is correlated with eye size and refractive state during myopia and hyperopia induction in chick. Thus, although responses at the single gene level are primarily distinct, larger transcript networks show extensive subtle bidirectional expression shifts.

The types of pathways implicated, as well as their correlation with eye size and refraction, suggest a close link with the morphological phenotypes of myopia and hyperopia. Expression of extracellular matrix gene sets was positively correlated with eye size and negatively correlated with refraction across lens groups on day 1. Additional tight junction and cell contraction pathways were positively correlated with ocular axial length only. The proteins encoded by these genes play a fundamental role in tissue morphogenesis (Heisenberg and Bellaïche, 2013) and the osmotic stress response (Brocker et al., 2012). These findings are consistent with a wide range of studies detailing changes in cell growth (Teakle et al., 1993; Troilo et al., 1996; Beresford et al., 1998), proliferation (Fischer and Reh, 2000; Tkatchenko et al., 2006), ionic concentrations (Seko et al., 2000; Liang et al., 2004; Crewther et al., 2006), and ion and water channel expression (Goodyear et al., 2008, 2010; Zhang et al., 2011) across the posterior eye during myopia induction. Structural changes during refractive error induction presumably alter the availability of metabolites from the choroidal blood supply (Shih et al., 1993), and the need for the metabolic production of energy and biosynthetic precursors to fuel cell growth (Lunt and Vander Heiden, 2011). Accordingly, expression of metabolic genes (primarily within fatty acid and mitochondrial metabolism pathways) was also positively correlated with ocular axial length and negatively correlated with refraction at all time-points (although proportional bidirectional shifts across both lens-groups were only seen on days 1 and 3). Finally, concurrent with the changes in fatty acid and structural pathway expression on day 1, apoptosis and immune pathways were positively correlated with eye length and negatively correlated with refraction. Although a role for immune processes in refractive compensation is less established, these findings are consistent with growing evidence in the refractive error field (Lazuk and Slepova, 1994; Mao et al., 2006; Mcglinn et al., 2007; Shelton et al., 2008; Long et al., 2013; Gao et al., 2015), as well as literature linking lipid metabolism and osmotic stress with immune and inflammatory responses (Daynes and Jones, 2002; Brocker et al., 2012; Feske et al., 2015).

Our GSEA results are concordant with the findings of a recent study in mice, where differentially expressed proteins during myopia induction were enriched for cytoskeletal remodeling and cell adhesion processes, as well as unsaturated fatty acid beta-oxidation and oxidative phosphorylation metabolic pathways (Barathi et al., 2014). Proteomics studies have also implicated APOA1 (Bertrand et al., 2006), a lipid transport protein in the PPAR signaling cascade. At the transcriptome level, previous studies have linked a range of structural, metabolic, and immune pathways with ocular growth control (Tkatchenko et al., 2006; Brand et al., 2007; Mcglinn et al., 2007; Shelton et al., 2008; Stone et al., 2011), however our study is the first to identify bidirectional expression responses in these pathways during myopia and hyperopia induction.

Although our pathway findings are primarily novel, at the single gene level our results contain many commonalities with past studies. Here, we replicated past findings suggesting a role BMP and Wnt signaling in co-ordinating structural change (Mcglinn et al., 2007; Stone et al., 2011; Zhang et al., 2012; Ma et al., 2014). Indeed, *BMP2* was the only gene to show a sign-of-defocus dependent expression pattern in the present study. Genes mediated by BMP signaling (Nakanishi et al., 1997; Parisi et al., 2006; Inai et al., 2013; Chang et al., 2015) were also implicated, including *HAS2* and *PTX3* which were up-regulated during hyperopia induction and *CTGF* which was down-regulated during myopia induction. These findings are consistent with previous studies linking *CTGF* expression in retina/RPE (Mcglinn et al., 2007), and *PTX3* and hyaluronan expression in the choroid (Nickla et al., 1997; Summers Rada et al., 2010; He et al., 2014) with periods of altered ocular growth. *NPR3*, a gene that shows growth-specific expression shifts in the tree shrew sclera (Guo et al., 2014), was also up-regulated during hyperopia induction. Notably, *NPR3* has been associated with fluid transport across the RPE (Mikami et al., 1995; Dahrouj et al., 2013), while concurrent up-regulation of the inflammatory biomarker *PTX3* and hyaluronan promotes fluid accumulation in the extracellular space of other tissues (Day and De La Motte, 2005) suggesting that these three transcripts may play a role in choroidal expansion during hyperopia induction.

Genes associated with circadian and phototransduction processes were also differentially expressed during both myopia and hyperopia induction. These findings are similar to those of a past chick microarray study, where several circadian genes were differentially expressed during myopia induction (Stone et al., 2011). These previous findings contributed to interest in the role of circadian processes as an explanation for the effects of outdoor time on refractive development in children (Stone et al., 2013). Careful examination of our own data suggests that circadian and phototransduction gene expression changed during the slowing of growth in normally developing eyes. These same genes were differentially expressed when comparing lens wearing and no lens animals because lens wear (of both signs) accelerated the time-course of these developmental expression changes. Thus, the role of circadian genes in refractive compensation appears more complex than previously thought, and may be related to changes in photoreceptor functioning. As noted in the methods (Differential Gene Expression), the relatively small sample sizes used in the present study meant that the outcome of single gene analyses differed depending on the evaluation method used (see also Soneson and Delorenzi, 2013). In this context our single gene results should broadly be interpreted with caution, however, it should also be noted that the changes to phototransduction and circadian gene expression across all three groups were robust to the analysis approach used. Future studies are now needed to validate and further explore these single gene changes at the mRNA and protein level (e.g., using qPCR and Western blots).

As the ultimate goal of animal studies of ocular growth is to better understand the mechanisms underlying myopia development in human populations, we compared our findings in chick with the results of Genome-Wide Association Studies (GWAS) indexed in the NHGRI catalog (Welter et al., 2014). *BMP2* was the only single gene from our study to fall near myopia-associated SNPs (see Supplementary Table S8 for details). At the pathway level, however, the KEGG “Arrhythmogenic Right Ventricular Cardiomyopathy” gene set (which was positively correlated with axial length and negatively correlated with refraction on 1 day) has been previously linked with human myopia (Hysi et al., 2014a). More broadly, genes associated with refractive error in human populations have pleiotropic effects on non-ocular systems where they are strongly associated with systemic phenotypes including trans-fatty acid levels (Hysi et al., 2014b; Hysi, 2015). That both GWAS and transcriptome methodologies have implicated multiple (but different) genes within these ECM and fatty acid pathways suggests that the co-ordinated actions of broader biological networks are more functionally significant for growth control than single genes.

Although pathway analysis is a valuable tool for investigating ocular growth phenotypes, our novel findings suggest that the outcome of such investigations is strongly influenced by experimental design. Few past transcriptome wide studies have concurrently investigated myopia and hyperopia induction, and none have done so using GSEA. Instead, researchers have used analysis methods that associate networks or pathways with lists of differentially-expressed genes (e.g., Brand et al., 2007; Shelton et al., 2008; Stone et al., 2011). This approach suffers from poor sensitivity, especially when expression changes are subtle (Abatangelo et al., 2009; Bayerlová et al., 2015). Because GSEA does not require an arbitrary cut-off for differential gene expression it has a much larger functional range. This greater sensitivity makes GSEA particularly suited to refractive error datasets where expression changes are often modest [for example during hyperopia induction in the present–and previous (Stone et al., 2011)–studies]. However, GSEA is not without limitations; it shows poor specificity in some circumstances (Bayerlová et al., 2015) and (in the present study) the results are limited to the gene sets available in the KEGG database which has a particular focus on metabolic and signaling pathways (Bauer-Mehren et al., 2009; García-Campos et al., 2015). Future studies may benefit from using a wider range of databases and analysis methods. Regarding the latter, methods that incorporate pathway topology are promising but require further benchmarking (Bayerlová et al., 2015).

Our novel findings may also reflect the tissues profiled; like several previous transcriptome studies (Mcglinn et al., 2007; Shelton et al., 2008; Summers Rada and Wiechmann, 2009; Stone et al., 2011) we analyzed a combination of posterior ocular tissues. This systems-level approach proved useful in identifying expression shifts likely to be localized (such as sets of photoreceptor-specific genes) through to broader structural and metabolic shifts that presumably affect multiple cell-types. However, it has previously been shown that profiling multiple cell types can obscure localized expression changes, and that some components of the expression response during visually-regulated growth are reversed in sign across different ocular layers (Ashby and Feldkaemper, 2010; Penha et al., 2011). Thus, further studies are now needed to localize the expression shifts identified here to individual ocular layers, and to determine whether the bidirectional expression shifts observed play a direct role in mediating ocular growth and refractive change.

In conclusion, our data demonstrate that expression of genes in several structural, metabolic, and immune pathways is correlated with eye size and refraction across a spectrum of ocular growth conditions in chick (normal development, myopia induction, and hyperopia induction). These findings elucidate the transcriptional response underlying the broad morphological changes that occur in the retina, RPE, and choroid during refractive compensation. The involvement of metabolic and immune/apoptosis pathways suggests a further link between structural change and tissue health that may increase the vulnerability of myopic chick eyes to secondary pathologies (see Hayes et al., 1986; Liang et al., 1995, 2004; who describe signs of secondary pathologies in chick). Moreover, similar observations in slower primate models (Tkatchenko et al., 2006) and GSEA studies (see above) support the notion that these expression shifts may translate to human myopia where related biological processes [i.e., apoptosis (Xu et al., 1996), mechanical (Saw et al., 2005; Morgan et al., 2012) and oxidative stress (Francisco et al., 2015)] have been linked with the development of sight-threatening secondary disorders.

## Author contributions

NR, LG, NH, and SC conceived, designed and coordinated the study. NR collected the data. NR and NH conducted the data analysis. NR wrote and revised the manuscript with input from LG, NH, and SC.

## Additional information

Accession code: GSE78042.

### Conflict of interest statement

The authors declare that the research was conducted in the absence of any commercial or financial relationships that could be construed as a potential conflict of interest.
