# Supplementary material for: Bidirectional Expression of Metabolic, Structural, and Immune Pathways in Early Myopia and Hyperopia
Source: Front Neurosci. 2016 Aug 30;10:390. doi: 10.3389/fnins.2016.00390 (PMC5003873; doi:10.3389/fnins.2016.00390)
Supplement: Supplementary file 2 [file DataSheet2.PDF]

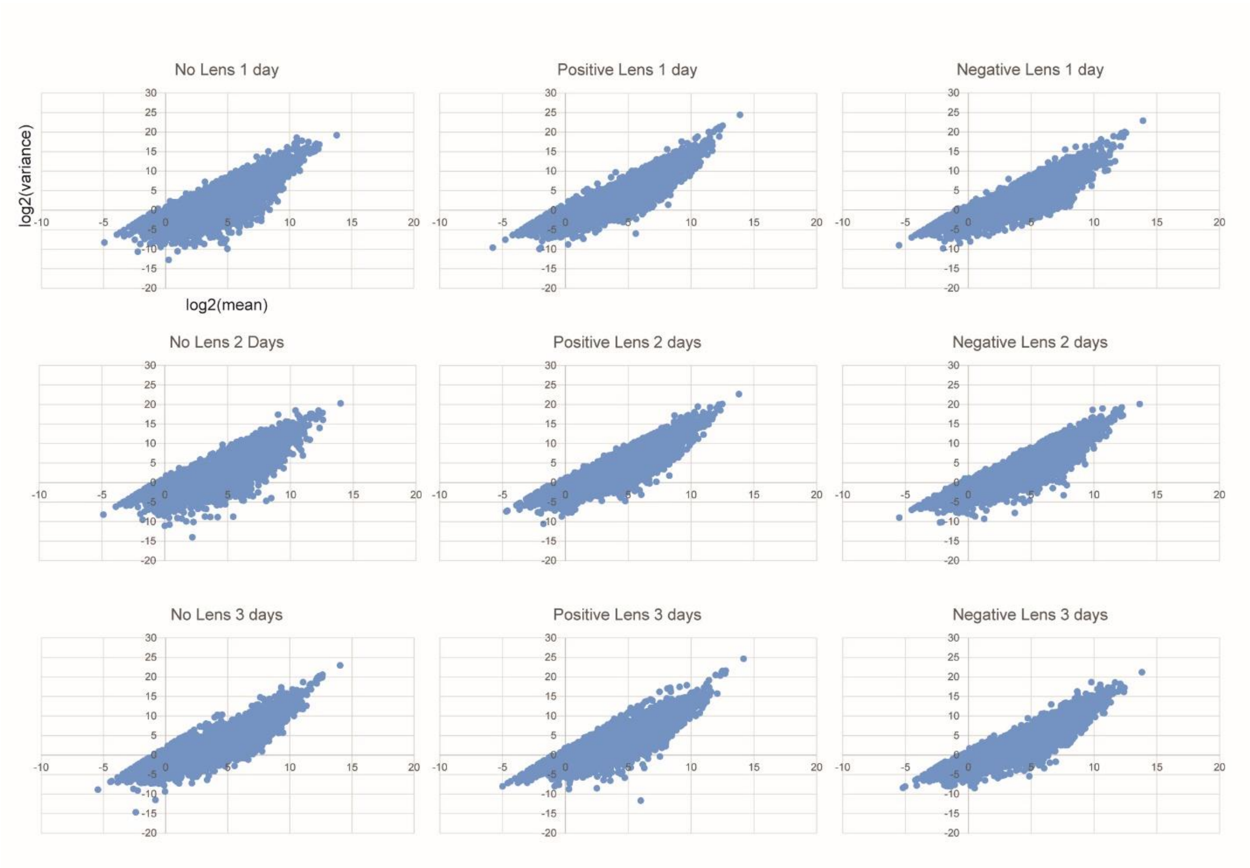

Supplementary Figure S1. Plots showing the mean to variance relationship for each sample group. Each dot represents the log2mean and log2variance for a gene. Note that variance is similar across groups.

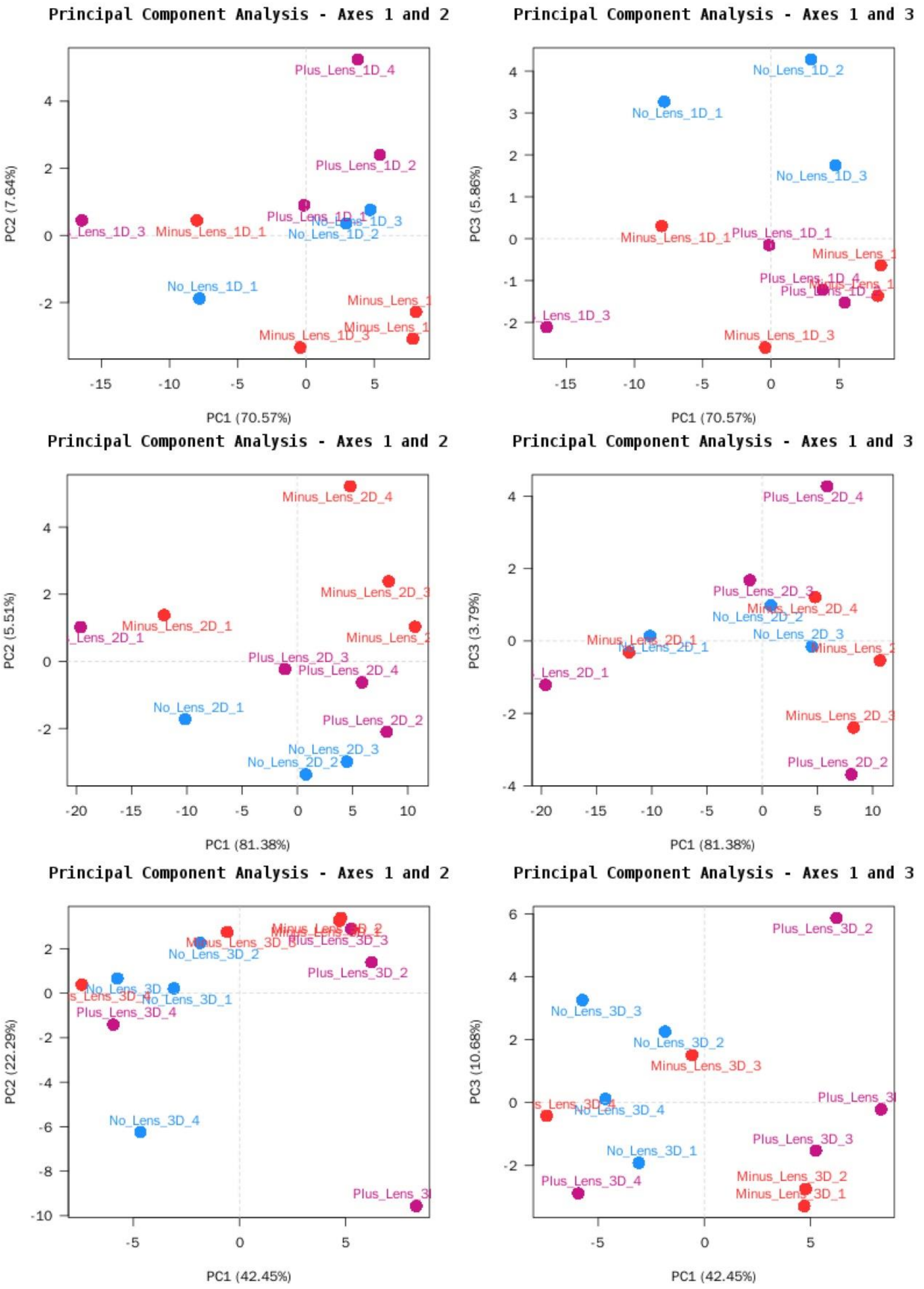

Supplementary Figure S2. Principal Component Analysis of samples at 1, 2 , and 3 days (labelled 1D, 2D, 3D) using Variance Stabilizing Transformation (VST).

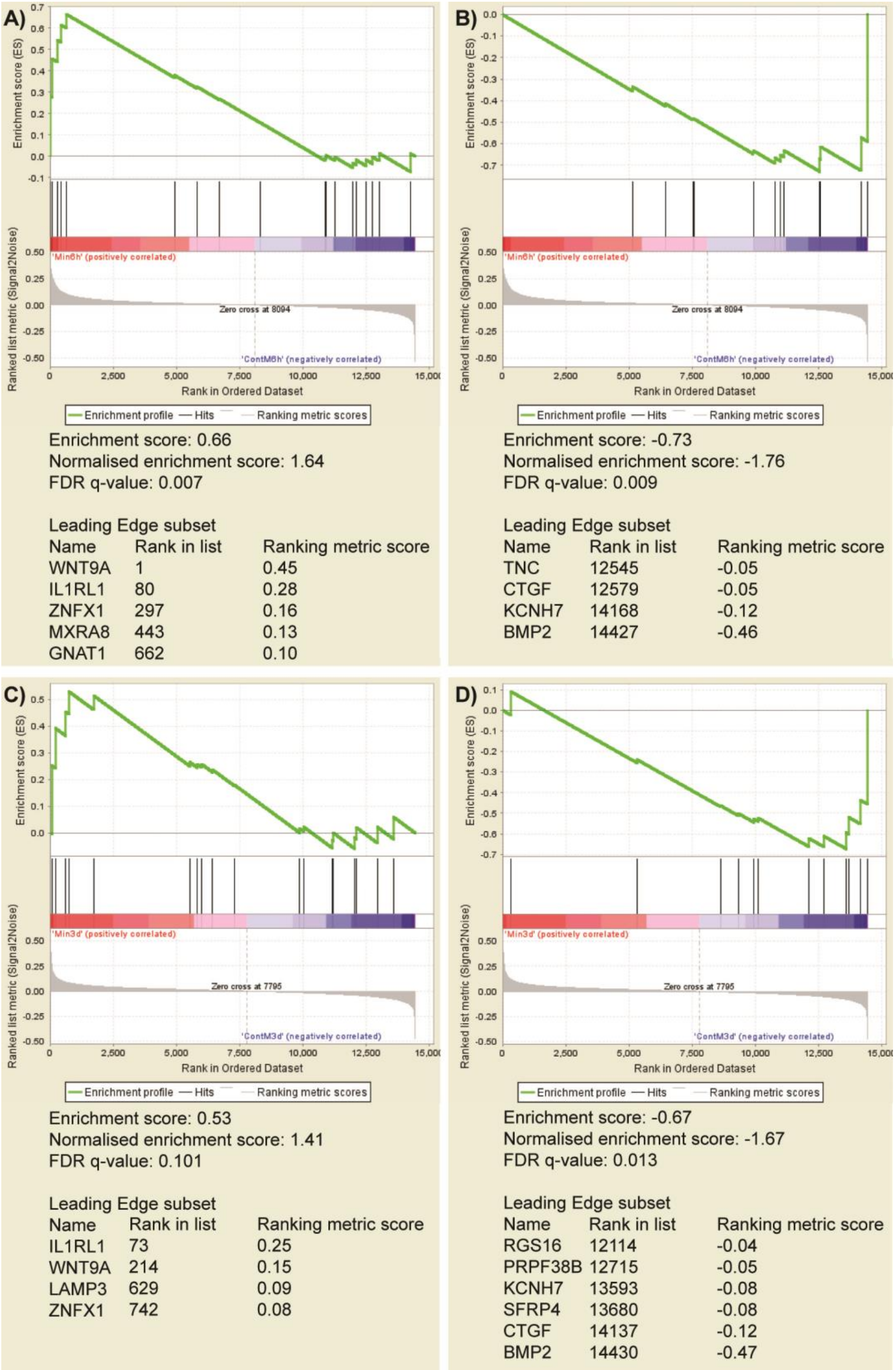

Supplementary Figure S3. Enrichment plots showing the rank of up- and down-regulated genes from our study in the most similar available microarray dataset (Stone, 2011). Up- and down-regulated gene sets were created from the list of genes differentially-expressed following 1, 2,

and 3 days of negative lens-wear (relative to age-matched No Lens chicks). These gene sets were tested for enrichment in Stone's 6 hour and 3 day negative lens data relative to fellow eye controls. Genes up-regulated during myopia induction in the present study were significantly enriched in Stone's (A) 6 hour and (C) 3 day negative lens datasets. Genes down-regulated during myopia induction in the present study were significantly enriched in Stone's (B) 6 hour and (D) 3 day fellow eye control datasets. Enrichment statistics and leading edge subset (LES) genes are provided under each plot. LES genes contribute most to the expression score (ES); for a positive ES (as in 'A' and 'C') the LES appear in the ranked list prior to the peak score, while for a negative ES (as in 'B' and 'D') the LES appear in the ranked list subsequent to the peak score. See Subramanian et al. (2005) for a detailed explanation of these statistics and plots.
